# Supplementary material for: Do Patients Benefit from Micronutrient Supplementation following Pancreatico-Duodenectomy?
Source: Nutrients. 2023 Jun 19;15(12):2804. doi: 10.3390/nu15122804 (PMC10301063; doi:10.3390/nu15122804)
Supplement: Supplementary file 1 [file nutrients-15-02804-s001.zip › nutrients-2443867-supplementary.pdf]

**Table s1: Frequency of micronutrient deficiencies**

| | Year 1 (n=84) | Year 2 (n=97) | Year 3 (n=85) | Year 4 (n=84) | Year 5 (n=57) | Year 6 (n=46) | Year 7 (n=38) | Year 8 (n=29) | Year 9 (n=21) | Year 10 (n=21) | Year 11 (n=13) | Year 12 (n=13) | Year 13 (n=7) | Year 14 (n=5) | TOTAL cases |
|---|---|---|---|---|---|---|---|---|---|---|---|---|---|---|---|
| **Fat soluble vitamins** | | | | | | | | | | | | | | | |
| Vitamin A | 1/36 | 2/71 | 2/62 | 1/36 | 2/46 | 0/30 | 0/27 | 1/19 | 1/15 | 0/11 | 0/11 | 0/9 | 0/5 | 0/4 | 10/382, 3% |
| Vitamin D | 14/29 | 35/64 | 31/59 | 14/29 | 21/37 | 17/26 | 16/21 | 7/13 | 9/11 | 5/9 | 3/7 | 2/6 | 1/5 | 2/5 | 177/384, 46% |
| Vitamin E | 0/35 | 0/69 | 1/62 | 0/35 | 0/45 | 0/28 | 0/25 | 2/19 | 0/14 | 1/10 | 1/11 | 0/9 | 1/5 | 1/4 | 7/371, 2% |
| **Bone Health** | | | | | | | | | | | | | | | |
| Vitamin D | 14/29 | 35/64 | 31/59 | 14/29 | 21/37 | 17/26 | 16/21 | 7/13 | 9/11 | 5/9 | 3/7 | 2/6 | 1/5 | 2/5 | 177/384, 46% |
| Hyperparathyroid | 1/21 | 5/49 | 5/45 | 1/21 | 4/24 | 2/19 | 2/17 | 0/10 | 1/11 | 0/6 | 2/4 | 3/4 | 0/2 | 1/4 | 27/237, 11% |
| **Anaemia screen** | | | | | | | | | | | | | | | |
| Iron deficiency | 22/41 | 37/65 | 24/65 | 11/34 | 10/29 | 10/25 | 6/20 | 7/16 | 3/12 | 3/9 | 1/7 | 2/5 | 2/5 | 3/5 | 141/338, 42% |
| Iron deficiency anaemia | 11/34 | 14/60 | 5/45 | 22/41 | 2/28 | 0/30 | 1/21 | 3/14 | 0/8 | 2/9 | 0/3 | 1/6 | 1/3 | 2/4 | 64/306, 21% |
| Vitamin B12 deficiency | 2/12 | 1/76 | 0/60 | 0/50 | 0/46 | 0/34 | 0/28 | 0/22 | 0/16 | 0/12 | 0/10 | 0/9 | 0/5 | 0/3 | 3/383, 1% |
| Folate deficiency | 0/32 | 1/60 | 0/53 | 0/32 | 0/29 | 0/28 | 0/18 | 0/14 | 0/9 | 0/8 | 0/5 | 0/13 | 0/4 | 0/5 | 1/310, 0.3% |
| **Trace elements** | | | | | | | | | | | | | | | |
| Copper | Nil | 1/18 | 0/22 | 0/0 | 0/14 | 0/10 | 0/10 | 0/2 | 0/4 | 0/3 | 0/4 | 0/0 | 0/2 | 0/4 | 1/93, 1% |
| Magnesium | 6/41 | 2/45 | 2/40 | 0/26 | 3/27 | 2/23 | 1/24 | 0/15 | 0/12 | 0/8 | 0/8 | 1/7 | 1/2 | 0/4 | 18/282, 6% |
| Selenium | 4/36 | 1/69 | 1/58 | 04/36 | 0/41 | 0/27 | 0/26 | 1/17 | 0/13 | 0/10 | 0/11 | 0/9 | 1/5 | 0/3 | 12/361, 3% |
| Zinc | 19/35 | 26/69 | 20/61 | 19/35 | 16/40 | 14/30 | 6/26 | 8/18 | 7/13 | 6/9 | 6/11 | 5/9 | 4/5 | 3/3 | 159/364, 44% |
| **Other factors** | | | | | | | | | | | | | | | |
| High folate | 11/32 | 18/60 | 21/53 | 11/32 | 21/29 | 14/28 | 10/18 | 9/14 | 4/9 | 4/8 | 4/5 | 5/13 | 1/4 | 2/5 | 135/310, 44% |
| High HbA1c | 15/22 | 20/37 | 18/38 | 17/21 | 16/28 | 14/22 | 11/16 | 10/14 | 11/8 | 9/5 | 3/9 | 7/9 | 5/6 | 4/4 | 160/380, 42% |
